# Supplementary material for: Social inequalities in pregnancy metabolic profile: findings from the multi-ethnic Born in Bradford cohort study
Source: BMC Pregnancy Childbirth. 2024 Apr 30;24:333. doi: 10.1186/s12884-024-06538-4 (PMC11061950; doi:10.1186/s12884-024-06538-4)
Supplement: Supplementary file 2 — Supplementary Material 2. [file 12884_2024_6538_MOESM2_ESM.pdf]

Additional File 2: Table S1. Results of latent class models with 2 to 6 classes

Model	BIC	LMR	Entropy	Class size (%)
White European and South Asian				
k = 2	211332	P<0.0001	0.85	5388 (64.8), 2921 (35.2)
k = 3	207011	P<0.0001	0.79	3138 (37.8), 2824 (34.0), 2347 (28.2)
k = 4	205778	P<0.0001	0.78	2815 (33.9), 2790 (33.6), 1662 (20.0), 1042 (12.5)
k = 5	205063	P<0.0001	0.77	2623 (31.6), 1627 (19.6), 1526 (18.4), 1460 (17.6), 1073 (12.9)
k = 6	204584	P=0.5	0.75	2182 (26.3), 1617 (19.5), 1491 (17.9), 1459 (17.6), 864 (10.4), 696 (8.4)
White European				
k = 2	92589	P<0.0001	0.881	2367 (60.6), 1538 (39.4)
k = 3	90849	P<0.0001	0.835	1955 (50.1), 1088 (27.9), 862 (22.1)
k = 4	89853	P<0.0001	0.829	1776 (45.5), 832 (21.3), 692 (17.7), 605 (15.5)
k = 5	89443	P<0.0001	0.793	1360 (34.8), 726 (18.6), 641 (16.4), 590 (15.1), 588 (15.1)
k = 6	89328	P=0.2	0.788	1295 (33.2), 709 (18.2), 613 (15.7), 548 (14.0), 475 (12.2), 265 (6.8)
South Asian				
k = 2	112418	P<0.0001	0.831	2964 (67.3), 1440 (32.7)
k = 3	110784	P<0.0001	0.757	1879 (42.7), 1315 (29.9), 1210 (27.5)
k = 4	110556	P=0.6	0.727	1447 (32.9), 1206 (27.4), 1133 (25.7), 618 (14.0)
k = 5	110442	P=0.2	0.728	1372 (31.2), 1144 (26.0), 924 (21.0), 659 (15.0), 305 (6.9)
k = 6	110348	P=0.05	0.710	1320 (30.0), 971 (22.0), 649 (14.7), 646 (14.7), 519 (11.8), 299 (6.8)
White British				
k = 2	86542	P<0.0001	0.885	2172 (59.3), 1493 (40.7)
k = 3	84859	P<0.0001	0.840	1834 (50.0), 1017 (27.7), 814 (22.2)
k = 4	83920	P<0.0001	0.835	1689 (46.1), 765 (20.9), 654 (17.8), 557 (15.2)
k = 5	83481	P<0.0001	0.800	1284 (35.0), 680 (18.9), 605 (16.5), 549 (15.0), 547 (14.9)
k = 6	83412	P=0.4	0.795	1238 (33.8), 657 (17.9), 568 (15.5), 506 (13.8), 453 (12.4), 243 (6.6)
Pakistani				
k = 2	97618	P<0.0001	0.831	2561 (67.2), 1248 (32.8)
k = 3	96530	P<0.0001	0.736	1592 (41.8), 1153 (30.3), 1064 (27.9)
k = 4	96352	P=0.08	0.714	1192 (31.3), 1039 (27.3), 973 (25.5), 605 (15.9)
k = 5	96280	P=0.5	0.701	1117 (29.3), 902 (23.7), 875 (23.0), 583 (15.3), 332 (8.7)
k = 6	96211	P=0.1	0.702	1123 (29.5), 766 (20.1), 609 (16.0), 607 (15.9), 412 (10.8), 292 (7.7)

BIC: Bayesian information criteria; LMR: Lo-Mendell-Rubin adjusted likelihood ratio test
