# Supplementary material for: Social inequalities in pregnancy metabolic profile: findings from the multi-ethnic Born in Bradford cohort study
Source: BMC Pregnancy Childbirth. 2024 Apr 30;24:333. doi: 10.1186/s12884-024-06538-4 (PMC11061950; doi:10.1186/s12884-024-06538-4)
Supplement: Supplementary file 3 — Supplementary Material 3. [file 12884_2024_6538_MOESM3_ESM.pdf]

Additional File 3: Table S2. Comparison of study participants with those excluded due to missing data on metabolic traits and gestational age.

	White European		South Asian	
Socioeconomic indicator	Included (n=3905)	Excluded (n=530)	Included (n=4404)	Excluded (n=877)
Index of Multiple Deprivation [No. (%)]				
Q1 (least deprived)	1947 (49.9)	249 (47.1)	960 (21.8)	178 (20.3)
Q2	1118 (28.6)	147 (27.8)	1638 (37.2)	345 (39.3)
Q3 (most deprived)	839 (21.5)	133 (25.1)	1805 (41.0)	354 (40.4)
Woman's education [No. (%)]				
<5 GCSE equivalent	733 (21.0)	112 (23.8)	1057 (25.3)	214 (25.9)
5 GCSE equivalent	1308 (37.5)	154 (32.7)	1296 (31.0)	279 (33.7)
A-level equivalent	675 (19.3)	79 (16.8)	568 (13.6)	105 (12.7)
Higher than A-level	776 (22.2)	126 (26.8)	1254 (30.0)	230 (27.8)
Baby's father's education [No. (%)]				
<5 GCSE equivalent	663 (24.1)	90 (25.1)	650 (18.9)	126 (18.3)
5 GCSE equivalent	1000 (36.4)	109 (30.4)	1016 (29.5)	215 (31.2)
A-level equivalent	470 (17.1)	72 (20.1)	418 (12.1)	73 (10.6)
Higher than A-level	615 (22.4)	88 (24.5)	1365 (39.6)	276 (40.0)
Woman's employment status [No. (%)]				
Currently employed	2572 (65.9)	334 (63.0)	1211 (27.6)	238 (27.2)
Previously employed	989 (25.3)	154 (29.1)	1269 (28.9)	313 (35.8)
Never employed	342 (8.8)	42 (7.9)	1915 (43.6)	324 (37.0)
Baby's father's employment status [No. (%)]				
Non-manual	1824 (50.8)	246 (50.5)	1433 (34.5)	264 (31.5)
Manual	1068 (29.7)	147 (30.2)	1622 (39.0)	354 (42.2)
Self-employed	368 (10.2)	46 (9.5)	825 (19.8)	158 (18.9)
Unemployed	332 (9.2)	48 (9.9)	279 (6.7)	62 (7.4)
Means tested benefit [No. (%)]				
Yes	1383 (35,5)	175 (33.1)	1911 (43.5)	376 (43.0)
No	2508 (64,5)	354 (66.9)	2478 (56.5)	498 (57.0)
How well mother and partner managing financially [No. (%)]				
Living comfortably	1036 (26.7)	150 (28.5)	1213 (27.7)	236 (27.1)
Doing alright	1607 (41.3)	195 (37.0)	1847 (42.2)	361 (41.5)
Just about getting by	973 (25.0)	143 (27.1)	981 (22.4)	201 (23.1)
Quite difficult or very difficult	272 (7.0)	39 (7.4)	333 (7.6)	73 (8.4)
Financial circumstance compared to year ago [No. (%)]				
Better off	1101 (28.3)	160 (30.3)	1308 (30.1)	254 (29.5)
Worse off	1007 (25.9)	137 (26.0)	717 (16.5)	155 (18.0)
About the same	1778 (45.8)	231 (43.8)	2315 (53.4)	453 (52.6)
Able to afford two pairs of all-weather shoes [No. (%)]				
Yes	3611 (96.1)	496 (96.3)	4269 (99.2)	846 (99.5)

No	145 (3.9)	19 (83.7)	33 (0.8)	4 (0.5)
Able to afford a small amount of money to spend on yourself each week [No. (%)]				
Yes	2966 (79.8)	408 (81.0)	3614 (86.5)	710 (85.5)
No	751 (20.2)	96 (19.1)	565 (13.5)	120 (14.5)
Able to afford to make regular savings of £10 a month [No. (%)]				
Yes	2697 (74.3)	364 (75.6)	3294 (80.8)	601 (77.2)
No	932 (25.7)	124 (25.4)	783 (19.2)	178 (22.9)
Housing tenure				
Owns outright	152 (4.2)	25 (5.0)	1068 (27.9)	197 (25.4)
Mortgage	1845 (50.7)	236 (47.3)	2020 (52.7)	427 (55.0)
Private landlord	1050 (28.9)	145 (29.1)	484 (12.6)	108 (13.9)
Social housing	590 (16.2)	93 (18.6)	261 (6.8)	44 (5.7)
Overcrowding (person per room) ([No. (%)]				
Q1	2548 (65.4)	328 (62.0)	1547 (35.2)	307 (35.1)
Q2	849 (21.8)	112 (21.2)	1393 (31.7)	294 (33.6)
Q3	500 (12.8)	89 (16.8)	1454 (33.1)	275 (31.4)
Up to date with bills [No. (%)]				
Yes	3369 (88.1)	454 (88.2)	3854 (91.4)	747 (89.1)
No	454 (11.9)	61 (11.8)	363 (8.6)	91 (10.9)
Able to afford to replace or repair major electrical goods [No. (%)]				
Yes	2485 (70.2)	339 (72.0)	3096 (79.2)	616 (78.8)
No	1057 (29.8)	132 (28.0)	813 (20.8)	166 (21.2)
Able to keep home warm enough in winter [No. (%)]				
Yes	3761 (97.8)	508 (96.6)	4175 (96.0)	822 (95.9)
No	84 (2.2)	18 (3.4)	172 (4.0)	42 (4.9)
Able to afford to replace any worn out furniture [No. (%)]				
Yes	2437 (69.1)	338 (70.7)	2830 (72.8)	560 (72.6)
No	1088 (30.9)	140 (29.3)	1059 (27.2)	211 (27.4)
Able to afford household contents insurance [No. (%)]				
Yes	2407 (84.6)	307 (84.8)	2184 (82.2)	407 (79.3)
No	438 (15.4)	55 (15.2)	474 (17.8)	106 (20.7)
Able to afford to keep home in decent state of decoration [No. (%)]				
Yes	3581 (94.3)	489 (95.0)	3743 (88.3)	740 (87.6)
No	215 (5.7)	26 (5.1)	497 (11.7)	105 (12.4)
