# Supplementary figures and images for: Social inequalities in pregnancy metabolic profile: findings from the multi-ethnic Born in Bradford cohort study
Source: BMC Pregnancy Childbirth. 2024 Apr 30;24:333. doi: 10.1186/s12884-024-06538-4 (PMC11061950; doi:10.1186/s12884-024-06538-4)

**Additional File 4: Figure S1. Distribution of metabolic traits by ethnicity group**

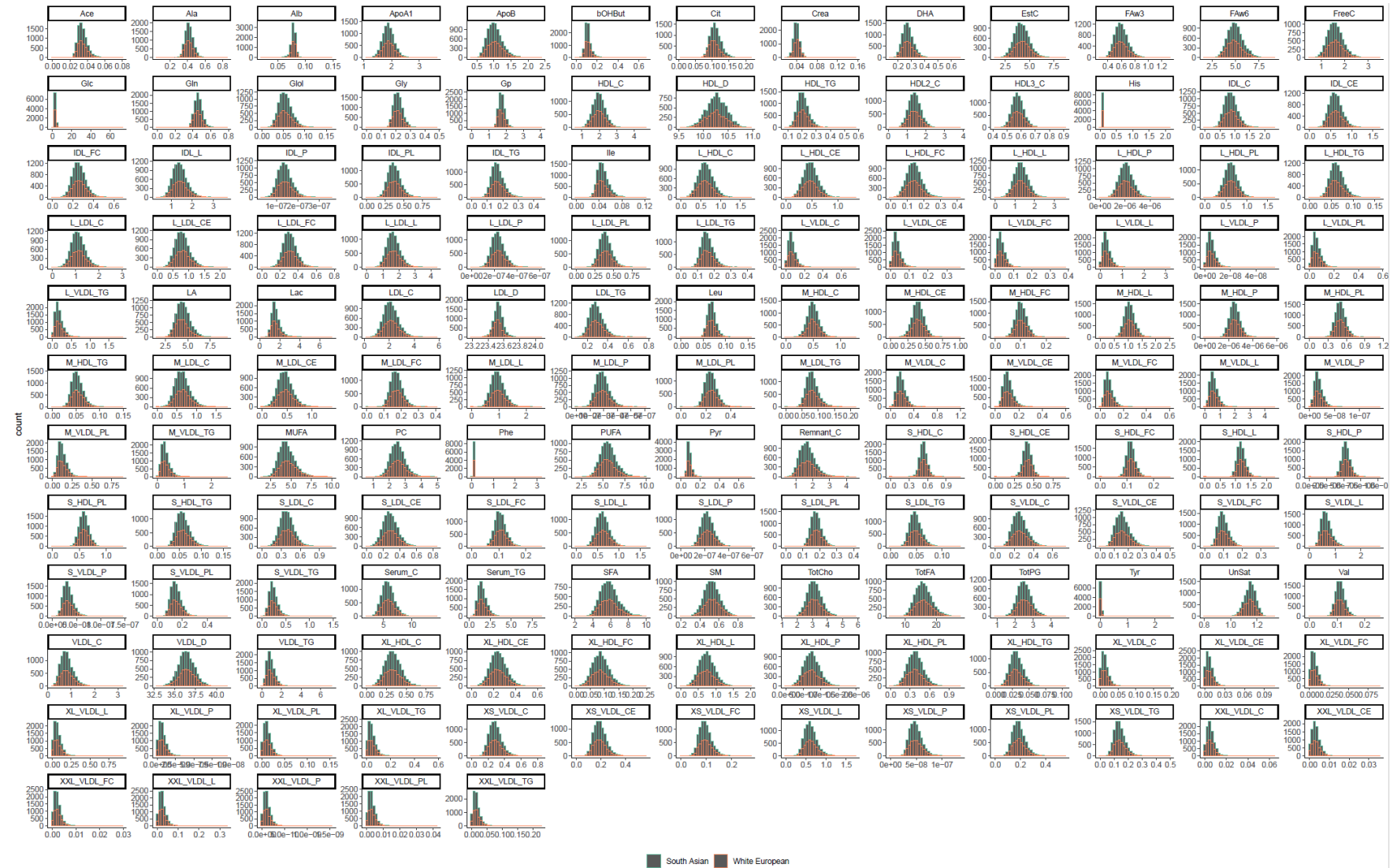

Supplement: Supplementary file 4 — Supplementary Material 4. [file 12884_2024_6538_MOESM4_ESM.pdf]
