# Supplementary material for: Social inequalities in pregnancy metabolic profile: findings from the multi-ethnic Born in Bradford cohort study
Source: BMC Pregnancy Childbirth. 2024 Apr 30;24:333. doi: 10.1186/s12884-024-06538-4 (PMC11061950; doi:10.1186/s12884-024-06538-4)

Additional File 9: Figure S2. Estimated mean probabilities for each SEP indicator variable in each ethnicity-specific SEP latent class sub-group in White British and Pakistani women.

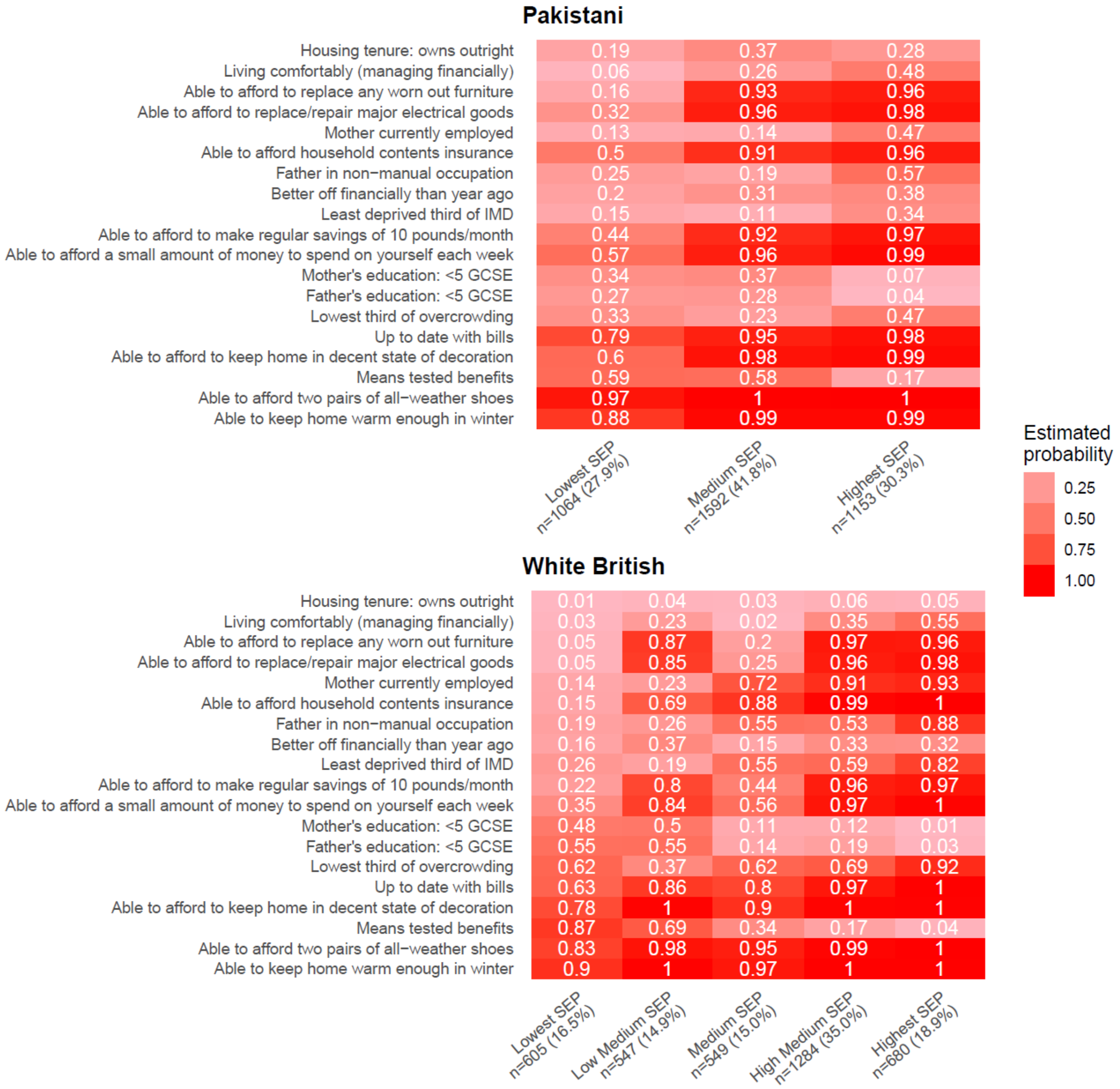

Supplement: Supplementary file 9 — Supplementary Material 9. [file 12884_2024_6538_MOESM9_ESM.pdf]
